# Double‐Shelled Phosphorus and Nitrogen Codoped Carbon Nanospheres as Efficient Polysulfide Mediator for High‐Performance Lithium–Sulfur Batteries

**DOI:** 10.1002/advs.201800621

**Published:** 2018-09-08

**Authors:** Jin Wang, Hao Yang, Zhen Chen, Lili Zhang, Jilei Liu, Pei Liang, Hui Yang, Xiaodong Shen, Ze Xiang Shen

**Affiliations:** ^1^ College of Materials Science and Engineering Nanjing Tech University Nanjing 210009 P. R. China; ^2^ Division of Physics and Applied Physics School of Physical and Mathematical Sciences Nanyang Technological University 21 Nanyang Link Singapore 637371 Singapore; ^3^ Institute of Chemical and Engineering Sciences A*STAR 1 Pesek Road Jurong Island 627833 Singapore; ^4^ College of Optical and Electronic Technology China Jiliang University Hangzhou 310018 P. R. China; ^5^ CINTRA CNRS/NTU/Thales UMI 3288, 50 Nanyang Drive Singapore 637553 Singapore

**Keywords:** lithium–sulfur batteries, double shelled nanospheres, carbon spheres, N,S‐codoping

## Abstract

Lithium–sulfur batteries are regarded as very promising energy storage devices due to their high energy density, low cost, and environmental friendliness; however, their insulating properties and the instability of sulfur‐based electrodes impede the practical applications of Li–S batteries. Here, a versatile strategy to synthesize double‐shelled nitrogen and phosphorus codoped carbon spheres (NPDSCS) as an efficient sulfur host for Li–S batteries is reported. With strong trapping, good affinity, high adsorption for polysulfides, and the bifunctional catalyzing for sulfur redox processes, the developed NPDSCS cathodes with a high S loading of 72.4% exhibit large specific discharge capacity of 1326 mAh g^−1^ at 0.1C, high Coulombic efficiency, good rate capability, and excellent cycling performance with a reversible capacity of 814 mAh g^−1^ at 1C after 500 cycles.

## 1 Introduction

Lithium–sulfur (Li–S) batteries have drawn considerable attention owing to their superior merits, including high theoretical energy density of 2567 W h kg^−1^ with natural abundance, environmental friendliness, and low cost.^1^, ^2^, ^3^ These advantages endow their great potential to power electric vehicles and intensive renewable energy storage, which require power sources with high energy and power density as well as long cycle life. However, there are still many obstacles to overcome for the realization of the practical application of Li–S batteries. One of the most notorious issue is the shuttle effect, originated from polysulfides dissolution into the liquid organic electrolyte and their migration between Li anode and cathode during charge/discharge processes, leading to the low utilization of sulfur materials and the poor reversibility.^4^, ^5^, ^6^ Another problem is the electric insulation nature of sulfur and the intermediate lithium polysulfides (LiPSs, Li_2_S*_n_*, 3 ≤ *n* ≤ 8) that impedes electron transport and leads to low utilization of active materials.^7^, ^8^, ^9^ Besides, the large volumetric variation between sulfur and Li_2_S induces the mechanical failure of the cathode during lithiation and delithiation, resulting in capacity fading.^10^, ^11^

To solve the above issues, one of the most efficient efforts is to encapsulate sulfur cathodes with rational designs of carbonaceous matrixes, such as micro/mesoporous carbons,^12^, ^13^, ^14^, ^15^ porous hollow carbon nanospheres,^16^, ^17^, ^18^ carbon nanofibers/nanotubes,^19^, ^20^, ^21^, ^22^, ^23^ and graphene.^2^, ^24^, ^25^ Among them, hollow carbon spheres (HCSs) have received much research interest by virtue of several advantages: (1) the unique hollow structure for hosting large amount of sulfur; (2) the large interior void space fraction for accommodating the large volume change; (3) sufficient contact area with the electrolyte for rapid electrolyte permeation and ion transport. Especially, double‐shelled HCSs could maximize the advantages of HCSs. The unique double shells could effectively confine a large amount of sulfur, inhibit the outward diffusion of polysulfides, and accommodate large volume change upon intensive cycling. Lou and co‐workers first reported novel double‐shelled hollow carbon nanospheres synthesized based on a SnO_2_‐templated method, when they are employed as the sulfur host exhibiting improved performance of sulfur cathodes.^26^ Zhou et al. synthesized nitrogen‐doped double‐shelled hollow carbon spheres (NDHCSs) using TiO_2_ and polymer cotemplated method.^18^ The sulfur is encapsulated by NDHCSs followed by graphene wrapping, demonstrating significantly improved electrochemical performance. The synthesis of double‐shelled HCSs has usually relied on soft/hard template‐based routes. Although hard‐templating method is one of the most controllable strategies to fabricate HCS, a new and effective methodology is the necessary prerequisite to the targeted double‐shelled HCSs.

In fact, nonpolar carbon can only provide a weak physical adsorption to polar LiPSs during the whole charge/discharge cycling. Recently, various host materials, such as polar inorganics (including SiO_2_, TiO_2_, Ti_4_O_7_, Fe_3_O_4_, Fe_2_O_3_, MnO_2_, C_3_N_4_, CoS_2_, MoS_2_, metal/covalent organic frameworks) have been intensively employed to adsorb the polar LiPSs through chemical bonding.^20^, ^27^, ^28^, ^29^, ^30^, ^31^, ^32^, ^33^, ^34^, ^35^, ^36^, ^37^, ^38^, ^39^, ^40^, ^41^, ^42^, ^43^ In addition to the polar inorganics, heteroatom‐doped carbon materials show great promise for boosting the performance of Li–S batteries because electron‐rich atoms can polarize the host carbon atoms and strengthen the chemical affinity for polysulfides, catalyzing the redox reactions of sulfur species to reduce electrochemical polarization and enhance the ionic conductivity of Li_2_S.^44^ Accordingly, various heteroatoms‐doped carbon materials (such as nitrogen‐doped various carbon^13^, ^18^, ^45^) have been designed as the matrixes for LSBs with excellent electrochemical performance. Especially, dual heteroatoms codoped carbonaceous materials (such as N/S, N/O, N/P) have been demonstrated with high electrical conductivity and strong binding energies for polysulfides due to the highly polarized carbon atoms. Manthiram and co‐workers developed N,S‐codoped graphene sponges with a strong bound interface between graphene and soluble lithium polysulfides, thereby contributing to the improved cycling performance.^46^ Xiong and co‐workers reported a nitrogen and oxygen dual‐doped nonporous carbonaceous material as a robust scaffold for sulfur cathode with efficient confinement, exhibiting excellent cycle stability and rate performance.^47^ Recently, Yu and co‐workers reported that N,P‐codoped carbon could efficiently trap polysulfides through the strong interaction between S and P.^48^ Also, P doping in the carbon framework plays an important role in improving the reaction kinetics, as it may help catalyze the redox reactions of sulfur species to reduce electrochemical polarization and enhance the ionic conductivity of Li_2_S. Therefore, the investigation into the mechanism of chemical binding between N,P‐codoped carbon and LiPSs is worthwhile for understanding the structural benefits based on heteroatom‐doped carbon hosts for Li–S batteries. Nonetheless, up to now, the mechanism of the chemical binding between N,P‐codoped carbon and LiPSs is still not explicit.

Here, we first present a facile route to synthesize double‐shelled HCSs and its N,P‐codoped carbon sphere (NPDSCS) counterparts, as highly efficient sulfur host for Li–S batteries. In most reported cases, the preparation of double‐shelled HCS often involves multistep reactions, including a template‐engaged and step‐by‐step reaction.^18^, ^26^ It is difficult to control the size and morphology of double‐shelled HCS due to these techniques with a dynamic equilibrium process. Compared with the multistep templating and coating strategies, double‐shelled structured template can overcome the above drawbacks with more controllable experimental parameters. We demonstrate for the first time that double‐shelled HCSs with controllable structures can be directly templating synthesized using double‐shelled SiO_2_ hollow capsules, which are precisely controlled by selectively etching solid silica@mesoporous silica nanospheres (sSiO_2_@mSiO_2_) in a mildly alkaline solution. Then, sulfur materials are encapsulated in NPDSCS and the resulting cathode is donated as NPDSCS‐S. Benefiting from the unique double‐shelled structure, the NPDSCS‐S electrode exhibits several advantages. (i) First, the unique double‐shelled carbon spheres provide sufficient empty space to make sure high sulfur loading of 5.8 mg cm^−2^ and accommodate large volume change of sulfur during the discharge/charge process. (ii) N,P‐codoped porous carbon shells ensure sulfur materials with high electrical conductivity, accelerate the electron transport, and benefit the electrochemical reaction kinetics of Li–S cells. (iii) N,P‐codoped porous carbon not only provides physical confinement for the diffusion of polysulfides, but also serves as polysulfide‐trapping centers through the strong chemical interaction with polysulfides. (iv) P doping in the carbon shells can help catalyze the redox reactions of sulfur species to improve the electrochemical reaction kinetics. The above‐mentioned virtues enable the cathode with enhanced specific capacities, ultralong cycling stability, and improved rate capability. Moreover, stable performance can be achieved for high sulfur loading electrodes (≈5.8 mg cm^−2^) with high sulfur content (72.4 wt%).

## 2 Results and Discussion

### 2.1 Synthesis and Characterization of NPDSCS‐S Composites

**Figure 1:**
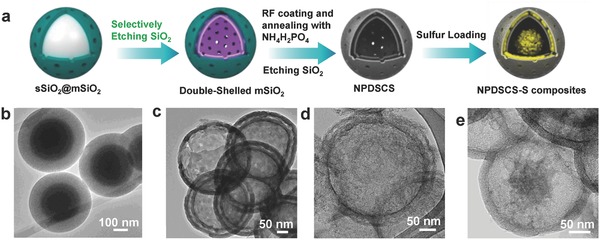
a) Schematic illustration of the fabrication process of NPDSCS‐S. TEM images of b) sSiO_2_@mSiO_2_, c) double‐shelled mSiO_2_, d) NPDSCS, and e) NPDSCS−S.

The synthetic strategy of NPDSCS is illustrated in **Figure** **1**a. First, the core–shell structured sSiO_2_@mSiO_2_ with an average diameter of around 450 nm are synthesized by a previously reported method (Figure 1b and Figure S1, Supporting Information).^49^ Second, double‐shell structured mSiO_2_ nanospheres (DSMS) with a diameter of ≈200 nm and a shell thickness of 50 nm are evolved from sSiO_2_@mSiO_2_ by a selective etching strategy in a mildly alkaline solution (Figure 1b and Figure S2, Supporting Information). Third, resorcinol formaldehyde is coated on DSMSs. Then, after mixed with NH_4_H_2_PO_4_ and carbonized in N_2_ atmosphere, and finally etched away SiO_2_, NPDSCS can be obtained, showing well‐maintained spherical morphologies with intact shells (Figure 1c and Figure S3, Supporting Information). Then, sulfur is impregnated into NPDSCS by a melt infiltration method, and the obtained NPDSCS‐S composites still maintain the original morphology (Figure 1d and Figure S4, Supporting Information).

**Figure 2:**
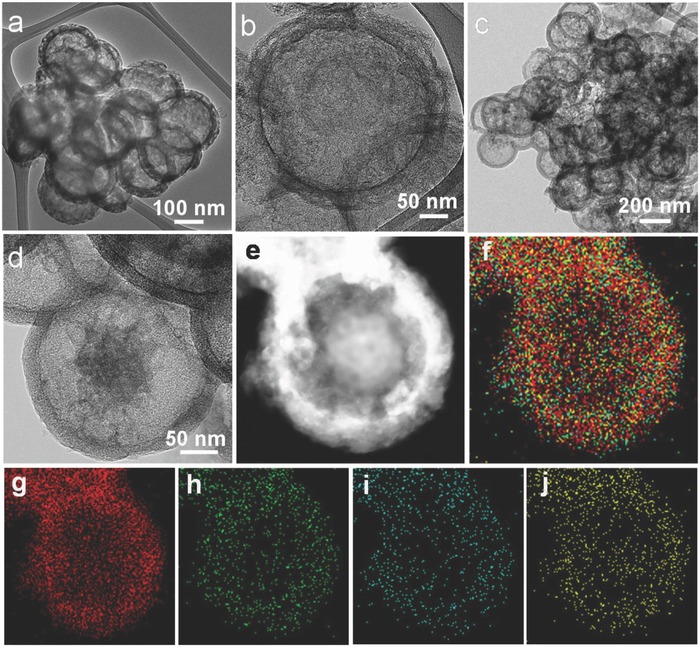
Morphology and structural characterization of NPDSCS and NPDSCS‐S. a,b) TEM image of NPDSCS. c,d) TEM image of NPDSCS‐S. e) STEM image and f) the overlapped elemental mapping image of NPDSCS‐S, g) carbon, h) nitrogen, i) phosphorus, and j) sulfur mapping images of NPDSCS‐S, indicating the homogeneous distribution of sulfur.

The morphology and microstructure of NPDSCS and NPDSCS‐S are further identified via transmission electron microscopy (TEM) observations. Uniform NPDSCS nanospheres exhibit double‐shelled structure with diameters from 200 to 300 nm and porous shell layers of around 50 nm (**Figure** **2**a,b). After sulfur impregnation, NPDSCS‐S still retains the original morphology. There are no sulfur agglomerations on the surface of NPDSCS and numerous particles with different contrast can be observed inside NPDSCS, indicating the nearly complete impregnation of sulfur into NPDSCS (Figure 2c). To investigate the sulfur microstructure and distribution in NPDSCS, high‐resolution TEM (HRTEM) and scanning TEM (STEM) images are examined. The dark area part in the core and shell of NPDSCS can be assigned to sulfur, which shows the opposite contrast in the STEM image (Figure 2e). The elemental mappings of carbon, nitrogen, phosphorus, sulfur, and their overlapped images can further demonstrate this result. Carbon, nitrogen, and phosphorus with similar signal intensities are uniformly distributed within NPDSCS and sulfur signal can be found within the shell and core (Figure 2f–j). For comparison, we also prepare double‐shelled N‐doped carbon nanospheres (NDSCS) as the sulfur host, which is prepared using polydopamine (PD) as the carbon‐coating layer (Figure S5, Supporting Information). Moreover, the double‐shelled carbon nanospheres (DSCS) are also investigated as the sulfur host.

**Figure 3:**
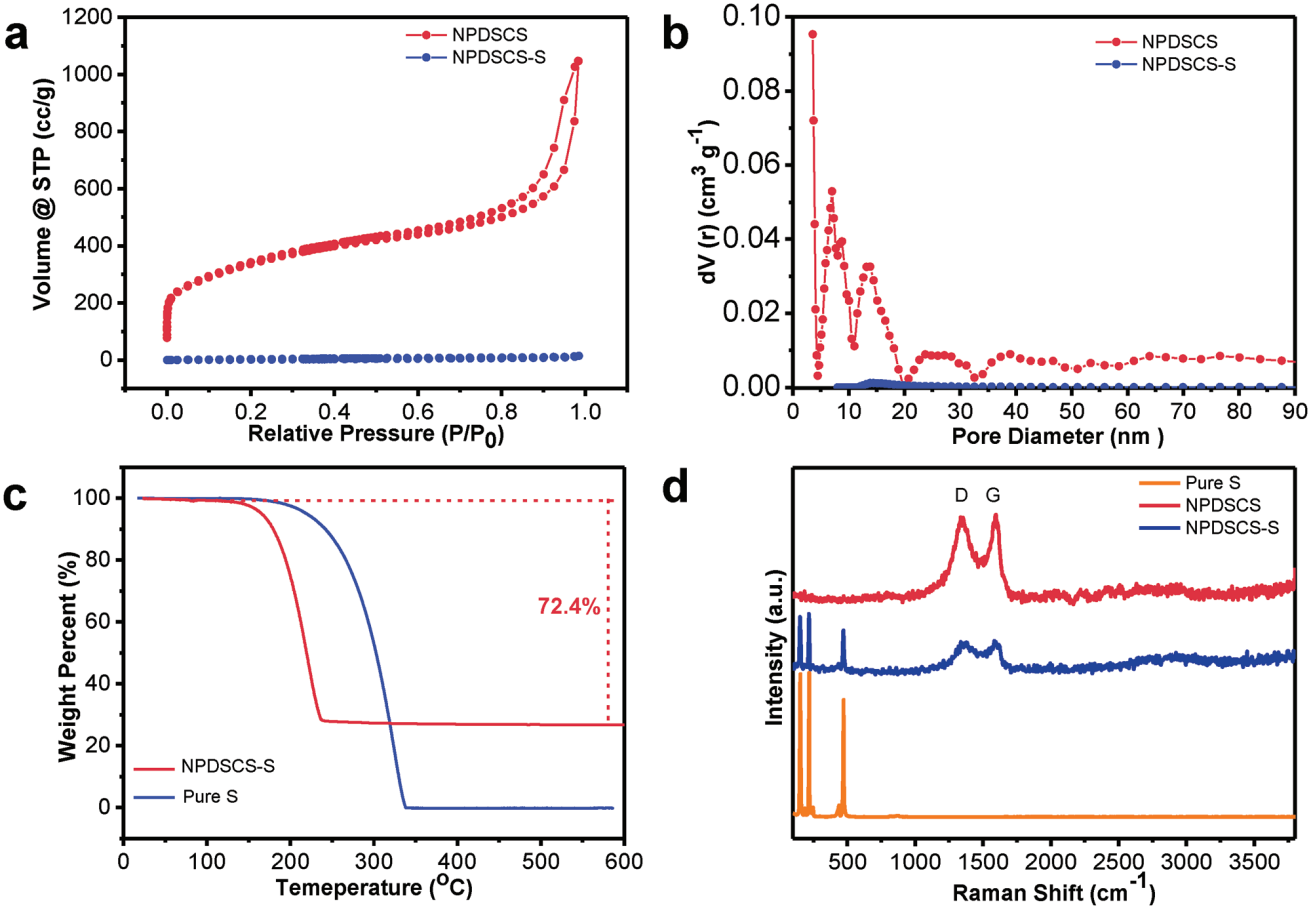
a) Nitrogen adsorption–desorption isotherms and b) pore size distribution curves of NPDSCS and NPDSCS‐S. c) TGA curve of pure S and NPDSCS‐S. d) Raman spectra of pure S, NPDSCS, and NPDSCS‐S.

To determine the specific surface area and pore size distribution of NPDSCS and NPDSCS‐S, full nitrogen adsorption–desorption isotherms are measured and shown in **Figure** **3**a,b. NPDSCS exhibits a high surface area of 1285.6 m^2^ g^−1^ and a large pore volume of 1.91 cm^3^ g^−1^. Nonetheless, surprisingly, the Brunauer–Emmett–Teller (BET) specific surface area of NPDSCS‐S decreases to 19.3 m^2^ g^−1^ and the pore volume drops to 0.24 cm^3^ g^−1^, indicating the successful impregnation of sulfur into NPDSCS. The peaks of the pore size distribution curves are located at 15 and 7 nm (Figure 3b). The high surface area and large pore volume can enable entire confinement of sulfur with high loading and efficiently alleviate the volume change of sulfur during cycling. Thus, NPDSCS is expected to be an ideal sulfur host with high chemisorption for lithium polysulfides.

The weight content and thermal stability of sulfur in NPDSCS‐S are measured by a thermogravimetric analysis (TGA), which is carried out from 30 to 600 °C at a heating rate of 10 °C min^−1^ in N_2_ atmosphere. As displayed in Figure 3c, a large weight loss of 72.4% can be observed between 200 and 350 °C resulting from the vaporization of sulfur, indicating the sulfur content of NPDSCS‐S is 72.4%. Raman spectra of NPDSCS‐S in Figure 3d showed characteristic peaks of both carbon (1360 and 1590 cm^−1^) and sulfur (below 600 cm^−1^), while NPDSCS only shows carbon signals. It should be noted that sulfur is completely impregnated in the core but shell parts as demonstrated in STEM images (Figure 2f). Therefore, Raman signals of sulfur can still be detected in NPDSCS‐S. *I*_D_/*I*_G_ ratios of NPDSCS‐S and NPDSCS are 0.98 and 1.10. The decreased *I*_D_/*I*_G_ ratio can be attributed to the graphitic domains with more defects in NPDSCS‐S due to the sulfur impregnation.

**Figure 4:**
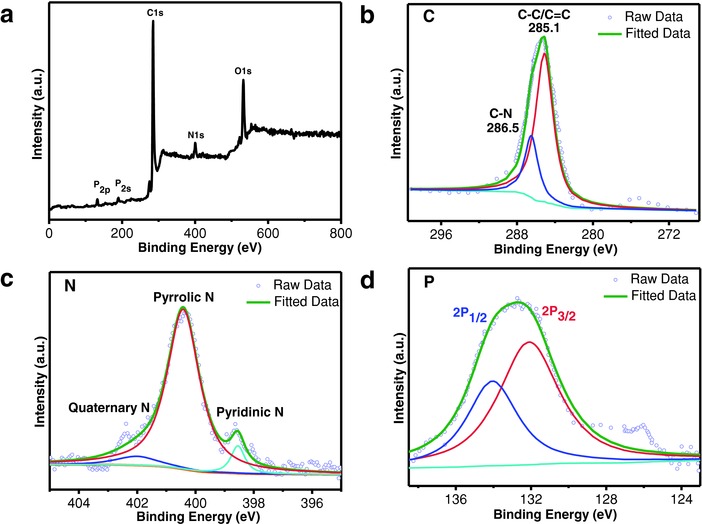
Surface composition analysis. a) XPS spectra of the surface chemical composition of NPDSCS. b) C 1s XPS spectrum. c) N 1s XPS spectrum. d) P 2p XPS spectrum of NPDSCS.

The chemical composition and surface chemical state of NPDSCS are further examined by X‐ray photoelectron spectroscopy (XPS), as shown in **Figure** **4**a. The peaks located at 132.7, 190.1, 285.2, 400.4, and 532.1 eV are observed, corresponding to P 2p, P 2s, C 1s, N 1s, and O 1s, respectively. From XPS spectra, the contents of N and P in N, P—C are estimated to be 3.3 and 2.8 at%, respectively. Figure 4b–d shows the deconvolution of high‐resolution peaks of C 1s, N 1s, and P 2p for NPDSCS. Two fitted peaks at 286.2 and 285.1 eV in C 1s spectrum can be ascribed to the C—N bond and conjugated C—C bonds (C—C/C=C) of sp^2^ hybridized carbon (Figure 4b). N 1s spectrum in Figure 4c can be fitted to three different peaks with binding energies of 398.2, 400.4, and 402.5 eV, corresponding to pyridinic, pyrrolic, and quaternary nitrogen atoms, respectively. The high‐resolution P 2p spectrum in Figure 4d can be deconvoluted into 2p 3/2 and 2p 1/2 peaks centered at 132.5 and 134 eV, which can be attributed to the interactions of P—C and P—O, respectively.^50^

### 2.2 The Electrochemical Performance of NPDSCS‐S Cathodes

**Figure 5:**
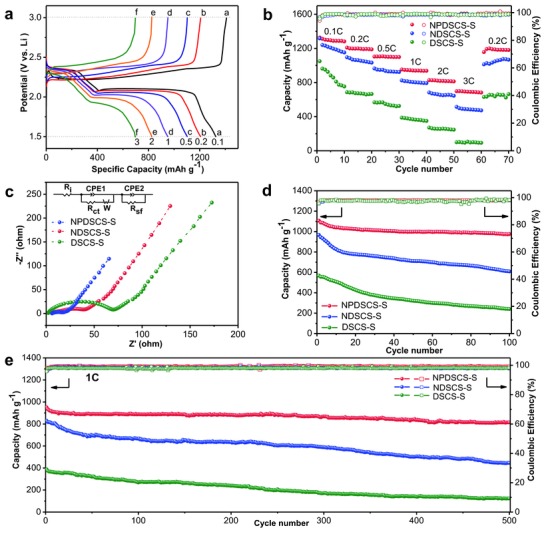
Electrochemical performance of the NPDSCS‐S, NDSCS‐S, and DSCS‐S composite electrodes for Li–S batteries. a) The first galvanostatic charge–discharge voltage profiles of the NPDSCS‐S electrode within a potential window of 1.5–3 V versus Li^+^/Li_0_. b) Discharge capacities of the NPDSCS‐S, NDSCS‐S and DSCS‐S electrodes at various current rates. c) Nyquist plots of the NPDSCS‐S, NDSCS‐S, and DSCS‐S electrodes, respectively. Cycling performance of the NPDSCS‐S, NDSCS‐S, and DSCS‐S electrodes for Li–S batteries at d) 0.5C for 100 cycles and e) 1C for 500 cycles.

To demonstrate the advantages of N, P‐codoped double‐shell structured carbon host in trapping LiPSs and stabilizing sulfur cathode, three NPDSCS‐S, NDSCS‐S, and DSCS‐S electrodes are employed as cathodes and paired with Li foil to fabricate Li–S coin cells. **Figure** **5**a and Figure S6 (Supporting Information) compare the galvanostatic charge/discharge profiles of three cathodes at various rates within a potential window of 1.5‐2.8 V from 0.1 to 3C. The NPDSCS‐S electrode shows two well‐defined discharge/charge plateaus, which can be ascribed to the reduction of elemental sulfur (S_8_) to long‐chain LiPSs and then to the short‐chain Li_2_S_2_/Li_2_S. The long oxidation plateau during the charge process can be attributed to a series of reactions from sulfides to polysulfides and finally to sulfur. With increasing the current densities, these plateaus still maintain well and exhibit low overpotentials (Δ*E*) between charge and discharge plateaus at high current rates (166 and 279 mV at 0.2 and 1C, Figure S6, Supporting Information), indicating an excellent kinetic reaction process. In contrast, NDSCS‐S and DSCS‐S exhibit sloping discharge/charge plateaus and larger overpotentials (e.g., 210 and 290 mV at 0.2C, together with 330 and 381 mV at 1C). Therefore, the lower overpotential in NPDSCS‐S can be ascribed to the catalytic effect of N,P—C on the redox reaction.^41^, ^48^, ^51^, ^52^ The reversible capacities of NPDSCS‐S gradually decrease from 1326 to 1103, 951, and 697 mAh g^−1^ with increasing the current rates from 0.1 to 0.5, 1, and 3C as shown in Figure 5b, testifying its excellent rate capability. The NDSCS‐S and DSCS‐S electrodes exhibit much lower capacities of 1093 and 685 mAh g^−1^ at 0.2C, and much poorer rate capability (515 and 99 mAh g^−1^ at 3C). The specific capacity of NPDSCS‐S could recover to a stable capacity of 1156 mAh g^−1^ when the current density is abruptly switched back to 0.2C. These results evidently demonstrate that NPDSCS‐S can not only favor fast electron transfer to enhance the rate capability, but also efficiently trap LiPSs to promote the cycling stability. The electrochemical impedance spectroscopy (EIS) measurements are carried out to investigate the reaction kinetics of cathodes. The Nyquist plots of the NPDSCS‐S, NDSCS‐S, and DSCS‐S cathodes at the open‐circuit voltage before cycling are shown in Figure 5c. Obviously, the NPDSCS‐S cathode exhibits the smallest diameter of the semicircle in the high–medium frequency region, compared with NDSCS‐S and DSCS‐S cathodes, suggesting its lowest contact and charge‐transfer resistances. The equivalent circuit is illustrated in the inset of Figure 5c and the corresponding fitting results are listed in Table S1 (Supporting Information). Three samples show the same solution resistances due to the same electrolyte. However, the lowest charge transfer resistance of the NPDSCS‐S cathode well demonstrates a reduced polarization and enhanced electrochemical reaction kinetics.^53^

To further examine the structural advantages of NPDSCS‐S, the cycling performance of the cells with NPDSCS‐S, NDSCS‐S, and DSCS‐S cathodes is tested at 0.5C over 100 cycles (Figure 5d). The NPDSCS‐S electrode exhibits good cycling stability with a reversible capacity of 975 mAh g^−1^ after 100 cycles. In contrast, the NDSCS‐S and DSCS‐S electrodes only deliver reversible capacities of 609 and 241 mAh g^−1^, respectively. The NDSCS‐S electrode exhibits relatively poor cycling stability, but is still much superior compared to that of the bare DSCS‐S electrode (without N‐doped or N,P‐codoped carbon layer). This result demonstrates the NPDSCS‐S cathode can partly alleviate the dissolution of LiPSs into the electrolyte through the strong chemical interaction. For the practical application of Li–S batteries, good capacity retention at high current densities is essential. Figure 5e shows the NPDSCS‐S electrode over 500 cycles at 1C. For the NPDSCS‐S electrode, a discharge capacity of 814 mAh g^−1^ can be stabilized after 500 cycles, whereas the NDSCS‐S cathode fades from 830 to 433 mAh g^−1^ and similarly the DSCS‐S cathode fades from 388 to 123 mAh g^−1^. The NPDSCS‐S electrode exhibits the best cycling performance with a capacity fading rate of 0.029% cycle^−1^. The improved cycling and rate performance reveal that the unique double‐shelled nitrogen and phosphorus codoped carbon spheres with physical space confinement and strong chemisorption can help to immobilize polysulfides and efficiently suppress the polysulfide migration upon long‐term cycling.

### 2.3 Ex Situ Characterizations and Density Functional Theory (DFT) Calculations

**Figure 6:**
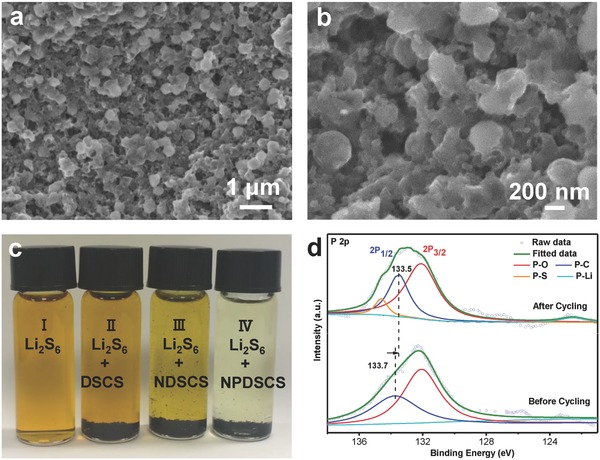
a,b) SEM images of NPDSCS‐S electrode after 100 cycles, indicating the good integrity of the electrode after long‐term cycling. c) Ex situ adsorption measurements (from the left to right, I: pure Li_2_S_6_, II: Li_2_S_6_ and SCS composites, III: Li_2_S_6_ + NDSCS composites, and IV: Li_2_S_6_ + NPDSCS composites). d) Elemental XPS spectra of P 2p for the NPDSCS‐S electrode before and after cycling.

In order to verify the origins of electrochemical results of the cells using the NPDSCS‐S, NDSCS‐S, and DSCS‐S electrodes, we examined various characterizations through scanning electron microscopy (SEM), XPS, and adsorption capability measurements. The electrodes are disassembled from Li–S batteries after 100 cycles, which is then washed with dimethyl ether (DME) to remove any residual LiTFSI. After cycling test, the spherical morphology and the intact microstructure of the NPDSCS‐S electrode are well preserved after intensive cycling (**Figure** **6**a,b). No obvious large sulfur agglomerates can be observed, implying the well‐defined NPDSCS‐S can effectively accommodate the large volume variation during cycling and also efficiently suppress the leakage of LiPSs, improving the electrochemical performance of NPDSCS‐S. Accordingly, XPS studies are further performed to investigate the underlying adsorption principles of NPDSCS‐S after cycling. Figure 6c shows the deconvolution of high‐resolution peaks of P 2p for NPDSCS‐S before and after cycling. Before cycling, P 2p spectrum showed two main peaks at 132 and 133.5 eV, which is assigned to the P—O and P—C bonds. After cycling, the P 2p spectrum has two additional peaks at 134.5 and 124.2 eV, corresponding to the interactions of P—S and P—Li, respectively. Besides, the P—C peak after cycling showed a negative chemical shift by 0.2 eV compared with that before cycling, indicating the reduced interaction of P—C bond due to new generation of P—Li bond. We also compare the N1s spectra for the NPDSCS‐S electrode before and after cycling as shown in Figure S7 (Supporting Information). After cycling, an additional peak at 399.7 eV is found in the N1s spectra for the NPDSCS‐S electrode, which may correspond to N—Li interaction. This result can well demonstrate the strong coupling of NPDSCS with LiPSs. Therefore, the adsorption experiments, SEM, and XPS can well demonstrate the high adsorption capability of the N,P‐codoped carbon host (NPDSCS) for LiPSs, which plays a vital role for high‐performance Li—S batteries. Adsorption capability plays an important role in Li—S battery performance for a sulfur host. In order to evaluate the adsorption ability of polysulfides on three sulfur hosts (NPDSCS, NDSCS, and DSCS), ex situ adsorption measurements are carried out as shown in Figure 6d. For the measurement, 5 mL of Li_2_S_6_ solution (5 mmol L^−1^) is used as a reference. Then, NPDSCS, NDSCS, and DSCS are ground into powders and immersed into Li_2_S_6_ solution for 12 h, and the color change is recorded. The Li_2_S_6_ solutions containing NPDSCS become almost colorless, and the color of the dissolved Li_2_S_6_ solution containing NDSCS fades slightly but still remains light yellow. Especially, the DSCS sample has no observable adsorption on the polysulfides since the color of the solution remains the same. The adsorption measurements remarkably demonstrate the strong adsorption of NPDSCS to LiPSs.

**Figure 7:**
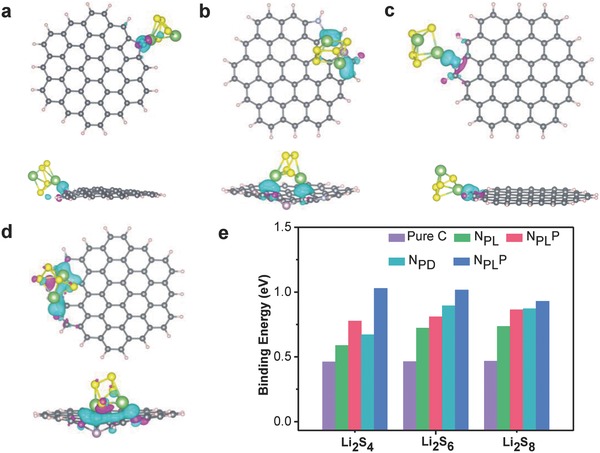
First‐principles calculation of carbon host‐sulfur containing guest interactions, showing the top views and side views of the optimized molecular configuration. The binding of the Li_2_S_4_ molecule with a) N_PL_, b) N_PL_P, c) N_PD_, and d) N_PD_P. Here, yellow, green, blue, purple, and pink spheres represent sulfur, lithium, nitrogen, phosphorus, and hydrogen atoms, respectively. e) DFT calculated binding energies between LiPSs and various heteroatom‐doped carbon.

To demonstrate the strong affinity between different components and sulfur species, theoretical simulation based on DFT method was also conducted to evaluate how the binding energy (*E*_b_) of LiPSs (including Li_2_S_4_, Li_2_S_6_, Li_2_S_8_) is influenced by the incorporation of N and/or P atoms into the graphene lattice. *E*_b_ is calculated to evaluate the binding strength between Li_2_S*_n_* clusters and perfect or doped graphene. It is defined as the energy difference between the adsorbed system (*E*_tot_) and the summation of individual Li_2_S*_n_* (*E*_s_) and perfect or doped graphene (*E*_sub_), and can express as

(1) $$E_{b}=E_{\mathrm{sub}}+E_{s}-E_{\mathrm{sub+s}}$$

where *E*_sub_, *E*_s_, and *E*_sub+s_ represent the total energies of carbon, an isolated sulfur‐containing cluster, and sulfur‐containing cluster binding to carbon. The van der Waals (vdW) interaction also contributes to the total binding energy as high as above 25%. Here, both the chemical bond and physical vdW interaction are considered when calculating the binding energy of LiPSs interacting with different heteroatom‐doped carbon. For N‐doped carbon, there are three common bonding configurations in the carbon lattice, including graphitic N (N_C_), pyridinic N (N_PD_), and pyrrolic N (N_PL_), which are also demonstrated by the XPS measurements (Figure 4d). In order to model the N,P‐codoped NPDSCS, two complex N,P‐codoping configurations with pyridinic N site (N_PD_P) and pyrrolic N site (N_PL_P) are investigated. **Figure** **7**a–d shows the geometries of the most stable adsorption configurations. The corresponding lithiation evolutions of binding energies are plotted in Figure 7e. As shown in Figure 7a–d, the binding energies of Li_2_S_4_ on N_PL_ and N_PD_ sites, which are 0.59 and 0.67 eV, are much lower than that for binding to the N_PL_P and N_PD_P sites, which are 0.78 and 1.03 eV. In addition, the binding energies of Li_2_S_6_ on the N_PL_P (0.72 eV) and N_PD_P (0.89 eV) sites are also much higher than that of N_PL_ (0.81 eV) and N_PD_ sites (1.02 eV), suggesting the strong chemisorption of LiPSs on the N,P‐codoping configurations. The binding energies of Li_2_S_8_ on the N_PL_P (0.73 eV) and N_PD_P (0.87 eV) sites are also much higher than that of N_PL_ (0.86 eV) and N_PD_ sites (0.93 eV). Besides, the binding energies of LiPSs interacting with the pristine carbon are obviously weaker, compared to various N and/or P‐doped carbon (Figure S8, Supporting Information). Thus, our DFT results clearly demonstrate that N,P‐codoped carbon (NPDSCS) can significantly enhance the binding of LiPSs species, when compared with the N‐doped carbon (NDSCS) and the pristine carbon (DSCS).

**Figure 8:**
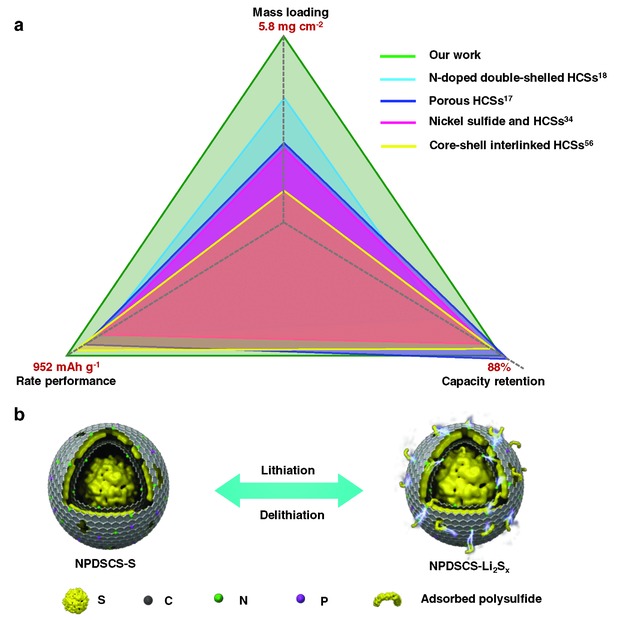
a) Electrochemical performance comparison with previous studies considering mass loading. All the capacity retention values were obtained at current densities of 0.5C after 100 cycles and the rate performance was evaluated at 1C. b) Schematic illustration of the trapping mechanism of sulfur and polysulfide species in NPDSCS‐S composites with physical block and chemical absorption.

To benchmark our result, the stereogram of rate performance and capacity retention against mass loading is plotted in **Figure** **8**a in comparison to previous work on hollow carbon electrodes (their values are also tabulated in Table S2, Supporting Information).^17^, ^18^, ^34^, ^52^, ^53^, ^54^ The comparison shows that our NPDSCS‐S electrode exhibits the best rate performance and also relatively high capacity retention despite the highest mass loading. The superior electrochemical performance of NPDSCS including improved sulfur utilization, good cyclic stability, and excellent rate capability can be attributed to the following synergistic effects (as illustrated in Figure 8b): First, the NPDSCS can provide sufficient empty space to host large amounts of sulfur and accommodate their volume change; Second, N,P‐codoped porous carbon shells ensure fast electron transfer; Third, N,P‐codoped NPDSCS serve as dual polysulfide‐trapping centers through physical and chemical confinement so that LiPSs can be largely anchored and retained inside the carbon spheres; In addition, porous shell/hollow core can make sure short lithium‐ion diffusion distance, enabling rapid ion transport and electrolyte diffusion; Besides, the hetero‐doped P atoms in NPDSCS act as a catalyst for the redox reactions of sulfur to improve the electrochemical reaction kinetics.

## 3 Conclusion

In summary, we have developed a versatile strategy to synthesize double‐shelled nitrogen and phosphorus codoped carbon spheres as an efficient sulfur host for Li–S batteries. The porous double‐shelled and hollow structure of NPDSCS provides sufficient empty space to host large amounts of sulfur to enhance the energy density, accommodate the volume variation, and suppress the dissolution of polysulfide intermediates. With strong trapping, good affinity, high adsorption for polysulfides, and the bifunctional catalyzing for sulfur redox processes, this NPDSCS has been demonstrated an efficient matrix for sulfur. DFT calculations and ex situ adsorption measurements can demonstrate the validity of the structural design in trapping LiPSs and enhancing the electrochemical performance. The NPDSCS‐S composites with a high S loading of 72.4% exhibit a large specific discharge capacity of 1326 mAh g^−1^ at 0.1C, high Coulombic efficiency, good rate capability, and excellent cycling performance with a reversible capacity of 814 mAh g^−1^ at 1C after 500 cycles. This work opens a new path to structured and molecular design based on heteroatom‐doped carbon hosts for Li–S batteries and should inspire further theoretical and experimental efforts on the nature of the multidoped carbon host for Li–S batteries.

## 4 Experimental Section

*Synthesis of sSiO_2_@mSiO_2_ and Double‐Shelled mSiO_2_*: Typically, 74 mL of ethanol, 10 mL of distilled water, and 3.14 mL of ammonium hydroxide were stirred together for 10 min at room temperature, and then 6 mL of tetraethylorthosilicate (TEOS) was rapidly added into the solution under magnetic stirring at 30 °C for 1 h, termed A. Then, the premixed solution with 1.093 g of hexadecyltrimethylammonium bromide (CTAB), 10 mL of ethanol, 20 mL of distilled water, and 3 mL of ammonium hydroxide was rapidly added into the solution A. The as‐prepared solution was stirred for 30 min, termed B. After that, 2 mL of TEOS was rapidly put into the B solution, and the magnetic stirring was lasted for another 6 h. The obtained sample sSiO_2_@mSiO_2_ was centrifuged and washed using deionized (DI) water and ethanol. The dried product (2 g) was uniformly dispersed in 0.6 m Na_2_CO_3_ (100 mL) under magnetic stirring at 80 °C for 1 h. The etched product was washed thoroughly with water to completely remove Na_2_CO_3_ until pH is neutral. Finally, the double‐shelled mSiO_2_ products were dried under vacuum and calcined at 550 °C for 6 h to remove the surfactant (CTAB).

*Synthesis of NPDSCS and Other Control Composites*: The resorcinol–formaldehyde (RF) polymer resin was coated on double‐shelled mSiO_2_ products as carbon precursor. The synthesis of RF polymer resin was performed as reported elsewhere.^55^ In a typical coating process, 200 mg of double‐shelled mSiO_2_ powders were dispersed in the mixed solvent of 40 mL of ethanol and 20 mL of water, and the mixture was ultrasonicated for 30 min to disperse thoroughly. Then, 0.20 g of resorcinol and 1.2 mL of aqueous ammonia were rapidly added into the above solution under magnetic stirring at 30 °C for 18 h. The obtained product was finally collected by centrifugation and washed with water for three times, which was then dried at 60 °C under vacuum. To get DSCS, the as‐obtained sample was calcined at 850 °C for 2 h with a heating rate of 2 °C min^−1^. To get NPDSCS, the as‐obtained sample was mixed with (NH_4_)_2_HPO_4_ with a mass ration of 1:2 and calcined at 850 °C for 2 h with a heating rate of 2 °C min^−1^. To get NDSCS, the as‐obtained sample (100 mg) was added into the mixed solution (80 mL) containing 200 mg of PEO‐PPO‐PEO (P123), 120 mg of dopamine, and 75 mg of 2‐amino‐2‐hydroxymethyl‐propane‐1,3‐dio (Tris) with continuous stirring in air for 24 h. After annealed at 800 °C for 2 h in flowing argon to carbonize polydopamine into nitrogen‐doped carbon.

*Synthesis of NPDSCS‐S and Other Control Composites*: NPDSCS‐S was obtained by a modified melt‐diffusion method. The NPDSCS was mixed sulfur powder with a weight ratio of 3:1 and sealed in a glass of vessel under argon protection. The mixture was heated at 155 °C for 12 h. For comparison, NDSCS‐S and DSCS‐S were also prepared, using the NDSCS and DSCS samples as the sulfur host by the similar preparation process, respectively.

*Cell Assembly and Electrochemical Measurements*: The working electrode was fabricated by mixing the 80 wt% active materials (NPDSCS, NDSCS, and DSCS), 10 wt% Super‐P, and 10 wt% polyvinylidene fluoride (PVDF). Sulfur loading in the NPDSCS electrode is as high as 5.8 mg cm^−2^ with high sulfur content of 72.4 wt%. CR 2032 coin cells were fabricated in an argon‐filled glove box using Celgard 2400 as the separator, lithium foil as the anode, and lithium bistrifluoromethanesulfonylimide LiTFSI (1 m in DOL/DME, 1:1 by volume) containing LiNO_3_ (2 wt%) as the electrolyte. The amount of electrolyte was fixed at 80 mL per cell. Galvanostatic charging and discharging tests were conducted using a battery tester (NEWARE) at different current rates. Galvanostatic charging and discharging tests were conducted using a battery tester (NEWARE) at different current rates. All the specific capacities reported in this work are based on the mass of sulfur only. Cyclic voltammetry (CV) was performed using an electrochemical workstation (CHI 760D, Chenhua, Shanghai) from 1.5 to 3 V at a scanning rate of 0.1 mV s^−1^. EIS was also carried out with an electrochemical workstation over a frequency range from 10^6^ Hz to 100 mHz at open‐circuit potential after two galvanostatic charging and discharging cycles at 100 mA g^−1^. For the cycled samples, the cycled cells were disassembled inside an Ar‐filled glove box, and the electrodes were rinsed with 1,2‐dimethoxyethane solvent to remove the lithium salt and dried inside the glove box at room temperature before analysis. For the ex situ adsorption measurement, Li_2_S_6_ solution was prepared by chemically reacting sublimed sulfur and Li_2_S in the blank electrolyte to form Li_2_S_6_ (1.0 m) in the solution. The solution was then stirred at 50 °C in an Ar‐filled glove box overnight to produce a brownish‐red Li_2_S_6_ catholyte solution.

*Characterization*: The morphology and structure characterization of the samples were carried out by TEM (JOEL JEM 2100F), field emission scanning electron microscopy (FESEM, Model JSM‐7600F, JEOL Ltd., Tokyo, Japan), and X‐ray diffraction (XRD, Bruke D8). Raman spectroscopy was recorded by Renishaw Raman Microscopy with 2.33 eV (532 nm) excitation laser. The XPS measurements were performed with a VG ESCALAB 220i‐XL system using a monochromatic Al Kα1 source (1486.6 eV). All XPS spectra were obtained in the constant pass energy (CPA) mode. The pass energy of analyzer was set to be 10 eV to have high measurement accuracy. The binding energy scale was calibrated with pure Au, Ag, and Cu by setting the Au 4f_7/2_, Ag 3d_5/2_, and Cu 2p_3/2_ at binding energy of 84.0, 368.3, and 932.7 eV, respectively. The surface area analysis was conducted using BET surface area analyzer (Micromeritics ASAP2020).

*Theoretical Calculations*: The atomic configurations and binding energies reported herein were calculated using DFT within the Perdew–Berke–Ernzerhof generalized gradient approximation (GGA‐PBE), as implemented in the Vienna Ab initio Simulation Package (VASP).^56^, ^57^, ^58^ For each monolayer substrate, a vacuum space of 20 Å was placed between adjacent layers to avoid periodic interactions. To model various heteroatom‐doping configurations at graphene edges and vacancies, a graphene flake consisting of 150 carbon atoms in a periodic simulation box of dimensions 35 × 35 × 20 Å was employed; dangling C bonds were passivated by H atoms. A 1 × 1 × 1 Gamma points *k*‐grid was used for sampling the Brillouin zones at structure calculation, whereas a denser mesh of 1 × 1 × 1 was used to calculate the densities of the states (DOS) for ensuring calculation accuracy. All the atomic positions and lattice vectors were fully optimized using a conjugate gradient algorithm to obtain the unstrained configuration. The electron wavefunctions were expanded in a plane wave basis with a kinetic energy cutoff of 400 eV. Atomic relaxation was performed until the change in total energy was less than 1 × 10^−4^ eV, and the maximum forces were smaller than 0.01 eV Å^−1^. The Fermi level shifted to zero for DOS.

## Conflict of Interest

The authors declare no conflict of interest.

## Supporting information

Supplementary
