# Supplementary material for: Double‐Shelled Phosphorus and Nitrogen Codoped Carbon Nanospheres as Efficient Polysulfide Mediator for High‐Performance Lithium–Sulfur Batteries
Source: Adv Sci (Weinh). 2018 Sep 8;5(11):1800621. doi: 10.1002/advs.201800621 (PMC6247042; doi:10.1002/advs.201800621)
Supplement: Supplementary file 1 — Supplementary [file ADVS-5-1800621-s001.pdf]

Copyright WILEY-VCH Verlag GmbH & Co. KGaA, 69469 Weinheim, Germany, 2018.

ADVANCED SCIENCE
Open Access

Supporting Information

for *Adv. Sci.,* DOI: 10.1002/advs.201800621

Double-Shelled Phosphorus and Nitrogen Codoped Carbon Nanospheres as Efficient Polysulfide Mediator for High-Performance Lithium–Sulfur Batteries

Jin Wang, Hao Yang, Zhen Chen, Lili Zhang, Jilei Liu, Pei Liang,* Hui Yang, Xiaodong Shen, and Ze Xiang Shen**

Supporting Information

Double–Shelled Phosphorus and Nitrogen Co–Doped Carbon Nanocapsules as Efficient Polysulfide Mediator for High–Performance Lithium–Sulfur Batteries

Jin Wang [a,b † *], Hao Yang [a †], Zhen Chen [b], Lili Zhang [c], Jilei Liu [b], Pei Liang [d *], Hui Yang [a], Xiaodong Shen [a], Zexiang Shen [a *]

[a] College of Materials Science and Engineering, Nanjing Tech University, Nanjing 210009, PR China

E–mail: msejwang@njtech.edu.cn (J. Wang)

[b] Division of Physics and Applied Physics, School of Physical and Mathematical Sciences, Nanyang Technological University, 21 Nanyang Link, 637371, Singapore

E–mail: Zexiang@ntu.edu.sg (Z.X. Shen)

[c] Institute of Chemical and Engineering Sciences, A*STAR, 1 Pesek Road, Jurong Island 627833, Singapore

[d] College of Optical and Electronic Technology, China Jiliang University, Hangzhou 310018, PR China

E–mail: plianghust@gmail.com (P. Liang)

[†] These authors contributed equally to this work

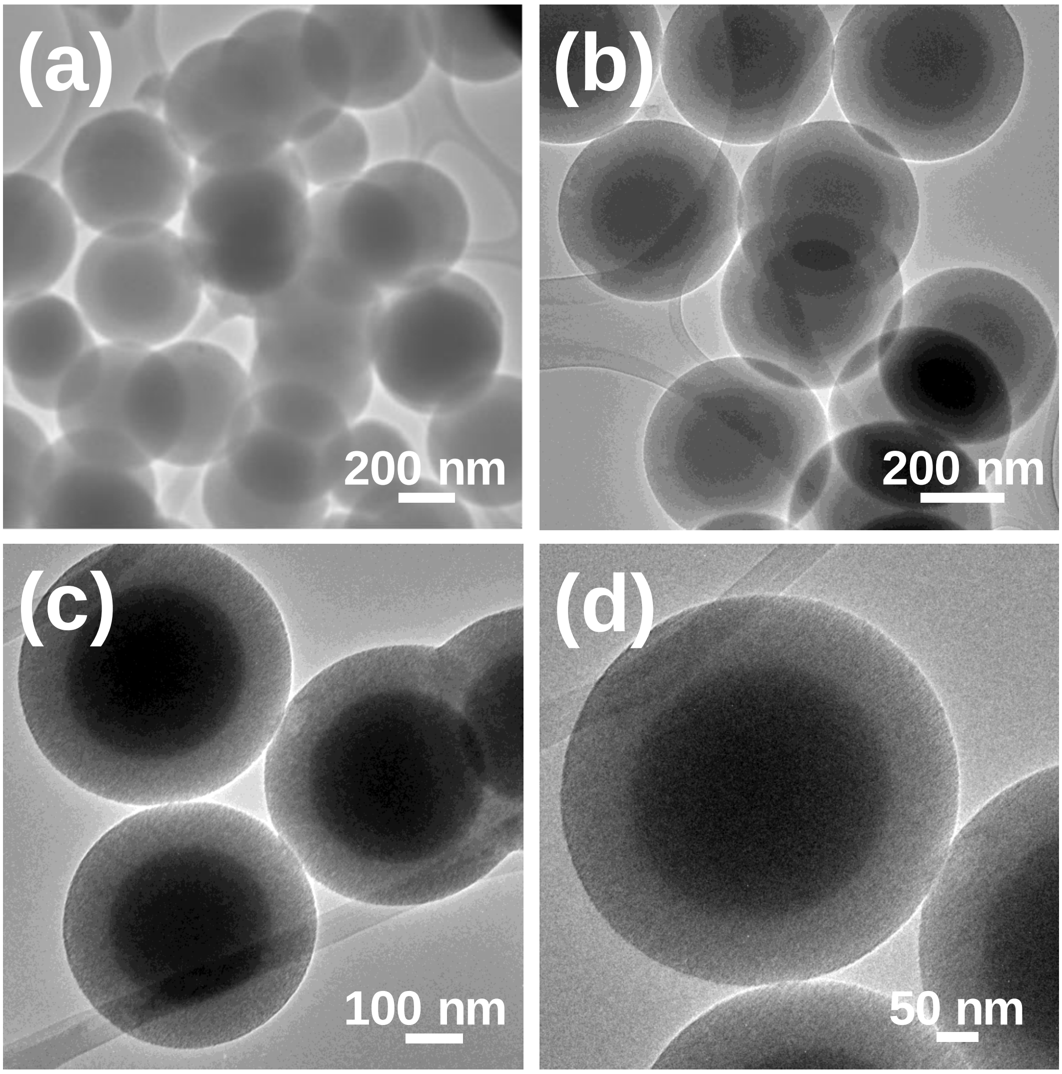

Figure S1 TEM images of $sSiO_2@mSiO_2$

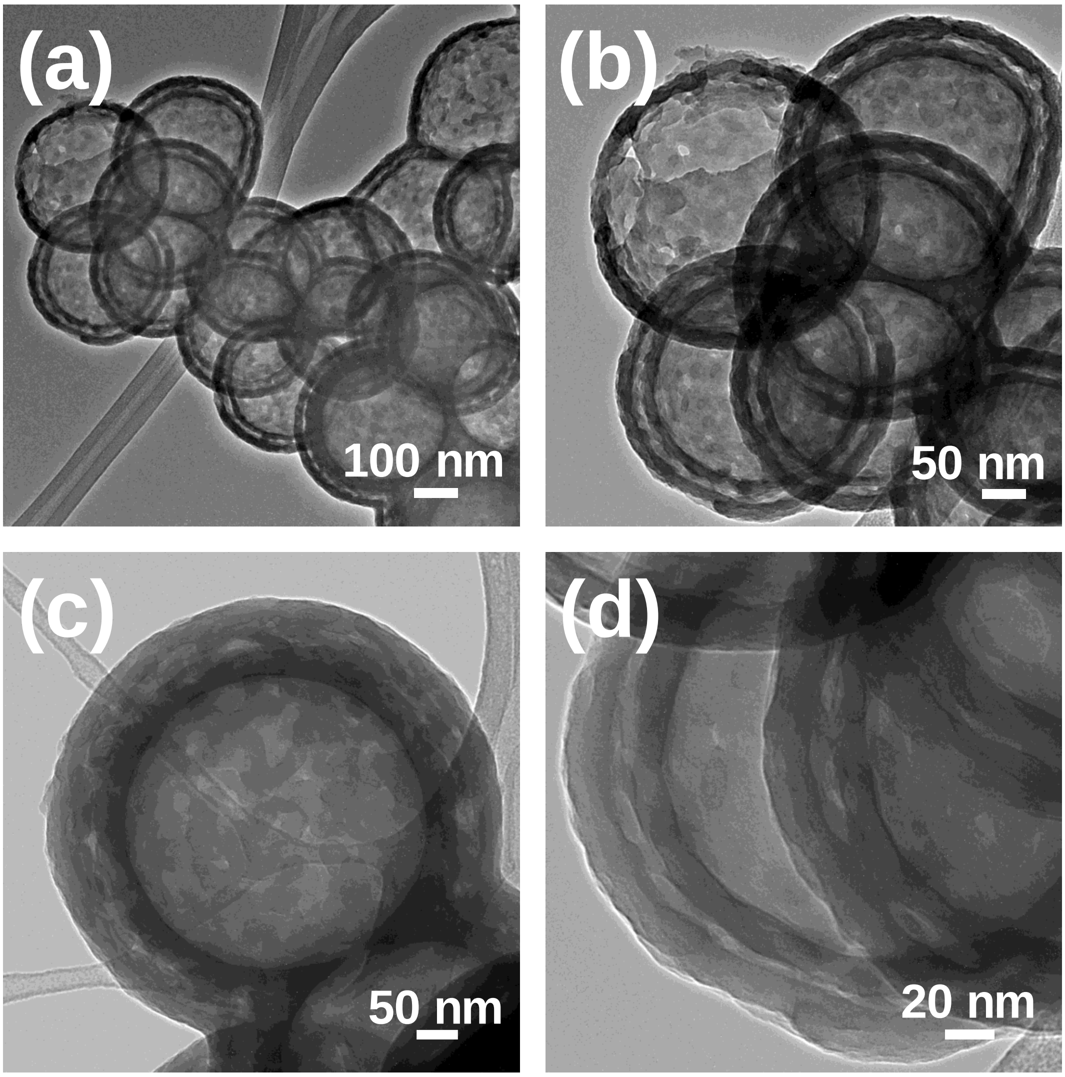

Figure S2 TEM images of double–shell structured $mSiO_2$ nanospheres (DSMS) prepared by etching $sSiO_2@mSiO_2$ in 0.6 M Na_2CO_3 solution for 0.5 h.

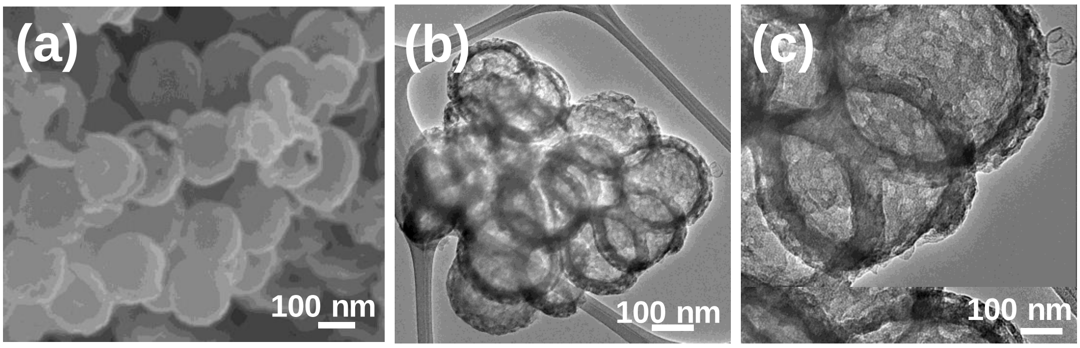

Figure S3 (a) SEM images, and (b) – (c) TEM images of double–shelled nitrogen and phosphorus co–doped carbon spheres

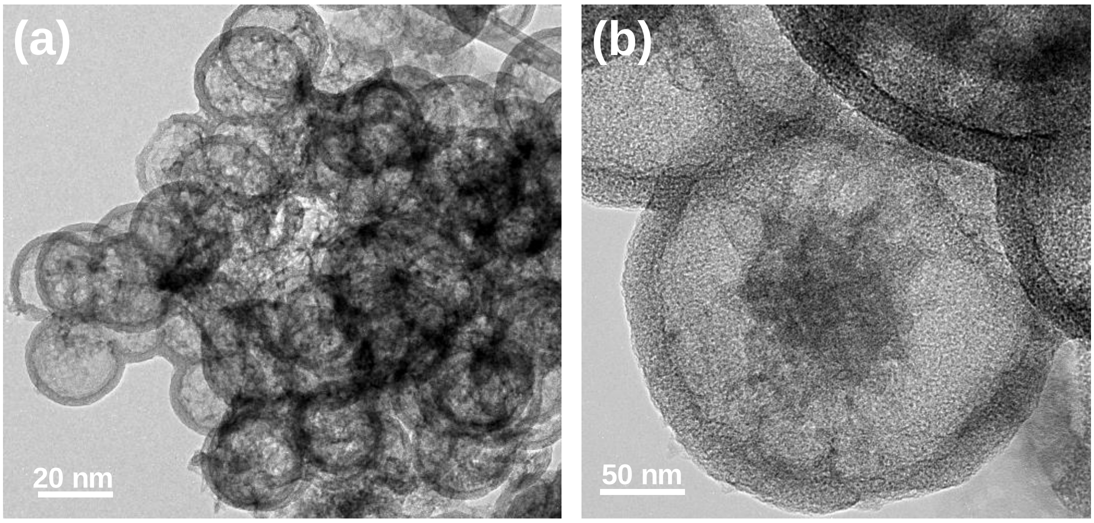

Figure S4 TEM images of NPDSC–S composites。

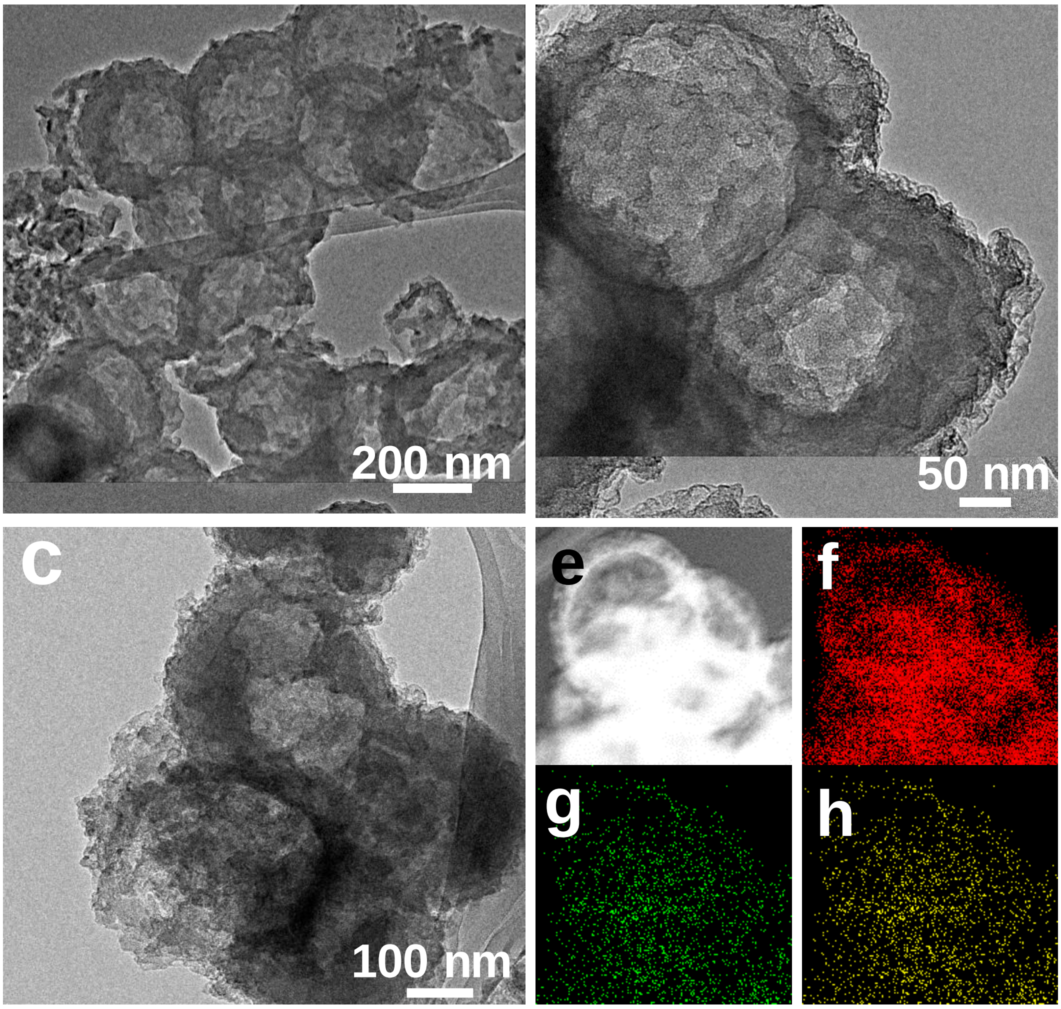

Figure S5 (a) and (b) TEM images of NDSC composites, (c) TEM image of NDSC–S composites, (e) Scanning transmission electron microscopy (STEM) image and the corresponding element mapping images of (f) carbon, (g) nitrogen, and (i) sulfur of NDSC–S composites, indicating the homogeneous distribution of all elements.

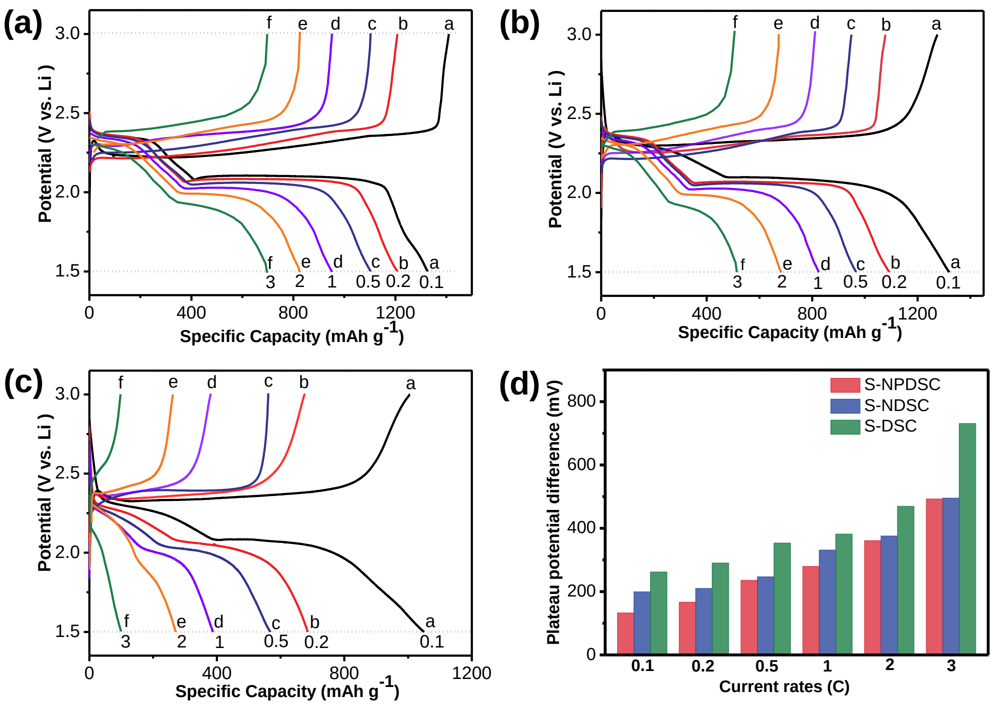

Figure S6 Galvanostatic charge–discharge voltage profiles of (a) NPDSC–S, (b) NDSC–S and (c) DSC–S electrodes at various current rates within a potential window of 1.5–3 V versus Li^{+}/Li_{0}.(d) Comparison of the potential difference between the charge and discharge plateaus at different current rates for the above three electrodes.

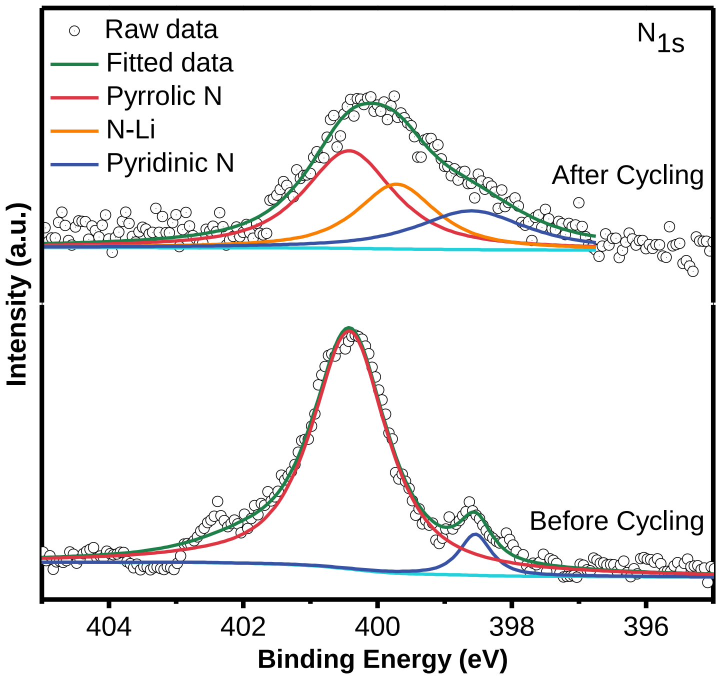

Figure S7 XPS spectra of N 1s in the NPDSCS–S electrode before and after cycling

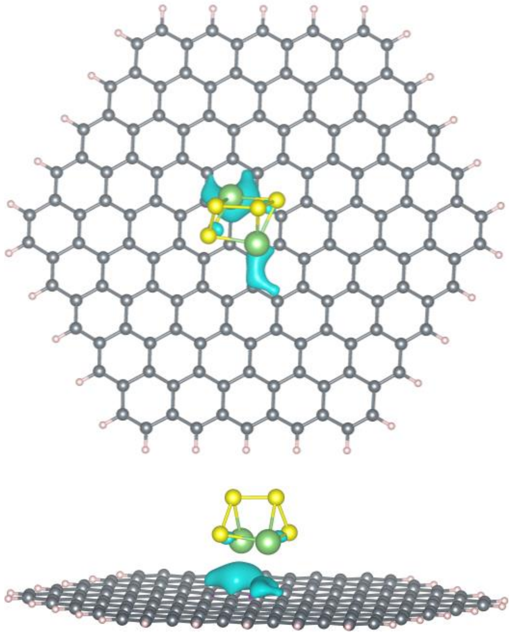

Figure S8 First–principles calculation of carbon host–sulfur containing guest interactions, showing the top views and side views of the optimized molecular configuration. The binding of the Li_2S_4 molecule with the pristine carbon.

Table S1. The fitted resistance parameters of SEI film (R_{sf}) and charge–transfer resistance (R_{ct}) for the NPDSCS–S, NDSCS–S and DSCS–S cathodes.

**Electrode description**	R_{sf} **(Ω)**	R_{ct} **(Ω)**
DSCS–S	68.5	139.3
NDSCS–S	46.4	68.7
NPDSCS–S	19.2	22.6

Table S2. The comparisons of hollow carbons for Li–S batteries.

Hollow carbons	S loading (mg cm^{2-})	Initial discharge capacity (mAh g^{-1}/C)	Decay rate (% per cycle/ cycling number)	Rate performance (mAh g^{-1}/C)
**Our work**	**5.8**	**1106/0.5C;**<br>**952/1C**	**0.12/100;**<br>**0.29/500**	**952/1C;**<br>**826/2C;**
Double-shelled hollow carbon spheres[1]	/	1020/0.1C	0.32/100	410/0.3C
Nitrogen-Doped Double-Shelled Hollow Carbon Spheres[2]	3.9	1360/0.2C	0.31/100	800/1C;<br>600/2C;
Porous hollow carbon spheres[3]	2-2.5	1015/0.2C	0.135/100	920/0.5C;<br>875/1C
Nitrogen-Doped Hollow Carbon Nanospheres[4]	0.5-0.7	1170/0.2C	0.12/100	920/0.5C;<br>720/1C;
Nickel Sulfide and Hollow Carbon Spheres[5]	2.3	1002/0.2C	0.14/200	723/0.5C

N-Doped Hollow Porous carbon bowls[6]	1.1-1.5	894/1C	0.053/400	516/4C
Hollow core-shell interlinked carbon spheres[7]	1.0	1100/0.5C	0.064/200	950/1C; 860/2C

Refercence:

1. Zhang, C.; Wu, H. B.; Yuan, C.; Guo, Z.; Lou, X. W., Confining Sulfur in Double-Shelled Hollow Carbon Spheres for Lithium–Sulfur Batteries. *Angewandte Chemie International Edition* **2012,** *51* (38), 9592-9595.
2. Zhou, G.; Zhao, Y.; Manthiram, A., Dual-Confined Flexible Sulfur Cathodes Encapsulated in Nitrogen-Doped Double-Shelled Hollow Carbon Spheres and Wrapped with Graphene for Li-S Batteries. *Advanced Energy Materials* **2015,** *5* (9), 1402263.
3. He, G.; Evers, S.; Liang, X.; Cuisinier, M.; Garsuch, A.; Nazar, L. F., Tailoring Porosity in Carbon Nanospheres for Lithium–Sulfur Battery Cathodes. *ACS Nano* **2013,** *7* (12), 10920-10930.
4. Zhou, W.; Wang, C.; Zhang, Q.; Abruña, H. D.; He, Y.; Wang, J.; Mao, S. X.; Xiao, X., Tailoring Pore Size of Nitrogen-Doped Hollow Carbon Nanospheres for Confining Sulfur in Lithium-Sulfur Batteries. *Advanced Energy Materials* **2015,** *5* (16), 1401752.
5. Ye, C.; Zhang, L.; Guo, C.; Li, D.; Vasileff, A.; Wang, H.; Qiao, S.-Z., A 3D Hybrid of Chemically Coupled Nickel Sulfide and Hollow Carbon Spheres for High Performance Lithium-Sulfur Batteries. *Advanced Functional Materials* **2017,** *27* (33), 1702524.
6. Pei, F.; An, T.; Zang, J.; Zhao, X.; Fang, X.; Zheng, M.; Dong, Q.; Zheng, N., From Hollow Carbon Spheres to N‐Doped Hollow Porous Carbon Bowls: Rational Design of Hollow Carbon Host for Li‐S Batteries. *Advanced Energy Materials* **2016,** *6* (8).
7. Sun, Q.; He, B.; Zhang, X.-Q.; Lu, A.-H., Engineering of Hollow Core–Shell Interlinked Carbon Spheres for Highly Stable Lithium–Sulfur Batteries. *ACS Nano* **2015,** *9* (8), 8504-8513.
